# Wound Infection with an Unusual Pathogen after Liver Transplantation

**DOI:** 10.1155/2020/8396507

**Published:** 2020-04-02

**Authors:** Sara Ghaderkhani, Zahra Ahmadinejad, Habibollah Dashti, Masoomeh Safaei, Fereshteh Ghiasvand

**Affiliations:** ^1^Department of Infectious Diseases, Imam Khomeini Hospital Complex, Tehran University of Medical Sciences, Tehran, Iran; ^2^Liver Transplantation Research Center, Department of Infectious Diseases, Imam Khomeini Hospital Complex, Tehran University of Medical Sciences, Tehran, Iran; ^3^Liver Transplantation Research Center, Imam Khomeini Hospital Complex, Tehran University of Medical Sciences, Tehran, Iran; ^4^Department of Pathology, Cancer Institute, Imam Khomeini Hospital Complex, Tehran University of Medical Sciences, Tehran, Iran

## Abstract

Mucormycosis is a rare and highly invasive fungal infection caused by Mucorales fungi of the class Zygomycetes. Cutaneous mucormycosis typically has a good survival rate when diagnosed early. In this report, we presented a patient with surgical site mucormycosis after liver transplant surgery. Our patient was a 50-year-old man with cirrhosis due to nonalcoholic steatohepatitis who received liver transplant from a deceased donor. On the 8^th^ day of transplant, the patient had fever and purulent discharge from the surgical site. The wound became black and necrotic in the next day. A microbiologic study showed mycelium in wound culture. The smear of the discharge was positive for aseptate hyphae, and the report of fungal culture revealed Rhizopus sp. In the histopathologic examination, mucormycosis was confirmed. The combination of antifungal and surgical debridement was a successful treatment in this case. Cutaneous fungal infections should be considered in the differential diagnosis of any nonhealing or black scar-infected wound that does not respond to broad-spectrum antibiotics.

## 1. Introduction

One of the invasive fungal infections involving skin and soft tissues is mucormycosis which is rare with low to high mortality [1–3]. This infection affects the rhino-orbital, respiratory, gastrointestinal, or cutaneous systems. These highly aggressive infections are often fatal; however, if mucormycosis is diagnosed early, it can be a treatable disease. The agents of Mucorales are widely spread in nature and could be found on spoiling vegetation and in soil. These organisms grow up rapidly and release huge numbers of spores that could become airborne. Mucorales can be present at any surfaces in hospital and also in atmosphere and water [4–6]. In this regard, all individuals have abundant exposure to these fungi during the daily routine events including hospitalized patients. Mucormycosis is a rare infection in healthy persons with a competent immune system. Nevertheless, this condition usually occurs in the presence of some underlying compromising condition. Cutaneous mucormycosis typically is a rare condition, with a good survival rate when diagnosed early [3, 4].

The purpose of this report is to describe the clinical course of a patient with surgical site infection with a mucosal agent, after liver transplantation.

## 2. Case Presentation

A 50-year-old man with cirrhosis due to nonalcoholic steatohepatitis (NASH) was admitted in the Hepatobiliary and Liver Transplantation Ward in Imam Khomeini Complex Hospital (one of the biggest LT centers in the Middle East), Tehran, Iran, for liver transplantation. He had hypertension (HTN) and fatty liver in his past medical history. Furthermore, the patient declared a history of gastrointestinal (GI) bleeding and encephalopathy in the time period of his disease. At the time of admission, the Model for End-Stage Liver Disease (MELD) score was estimated to be about 17 and the Child-Pugh score was eight. The Psychosocial Assessment of Candidacy for Transplantation (PACT) scale was 2 to 3. The laboratory findings of the patient before and early after LT are shown in Table 1.

Transplantation surgery was done without any serious complications, with the length of 5 hours. The technique of surgery was orthotopic transplantation from a deceased donor, and common bile duct (CBD) anastomosis was duct to duct. Also, not more than 2 unit packed cells were transfused. He received ampicillin sulbactam as an antibacterial prophylaxis for 48 hours after surgery. A day after surgery, the patient was routinely extubated and all physical exams were normal including the site of surgery. Daily Doppler sonography was normal. Antimicrobial prophylaxis with ganciclovir and fluconazole on day 1 and trimethoprim-sulfamethoxazole on the third day was started based on our center protocol.

Our center follows a local protocol for prophylaxis: targeted antifungal prophylaxis, preemptive therapy for Cytomegalovirus (CMV) except for high-risk patients including patients with acute liver failure, donor positive-recipient negative serology, and retransplantation that receive primary prophylaxis. We also use universal prophylaxis for Herpes virus (HSV) with acyclovir for the first 4 weeks after transplant and Pneumocystis pneumonia (PCP) with pyrimethamine-sulfadiazine for 6 months. In targeted antifungal prophylaxis, we assess the patients based on their risk factors; the high-risk patients include patients with retransplantation, reoperation within first month, and acute liver failure and those who need renal replacement therapy receive voriconazole for 4 weeks.

The patient with moderate risk received fluconazole until the prednisolone dose is 20 mg or less. The patients with prolonged time operation (more than 11 hours), blood transfusions more than 40 units, colonization with candidiasis in one month before transplant, bacterial infections, choledochojejunostomy technique for transplant, pulse therapy with corticosteroids, and antithymocyte globulin have moderate risk for invasive fungal infection, and others are at low risk that do not receive any antifungal prophylaxis.

**Figure 1:**
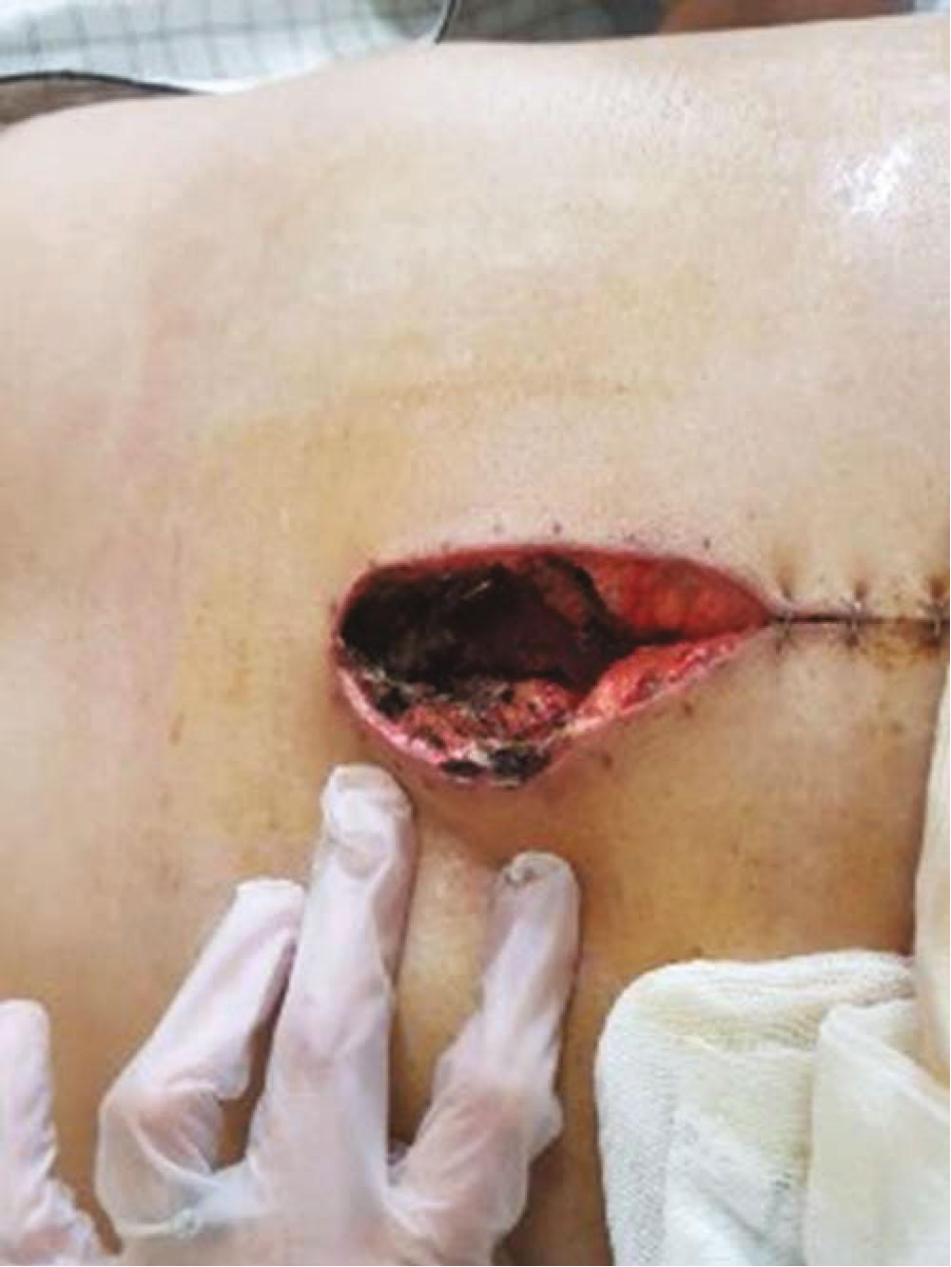
Wound appearance at the initial phase of disease (necrotic edge is seen).

**Figure 2:**
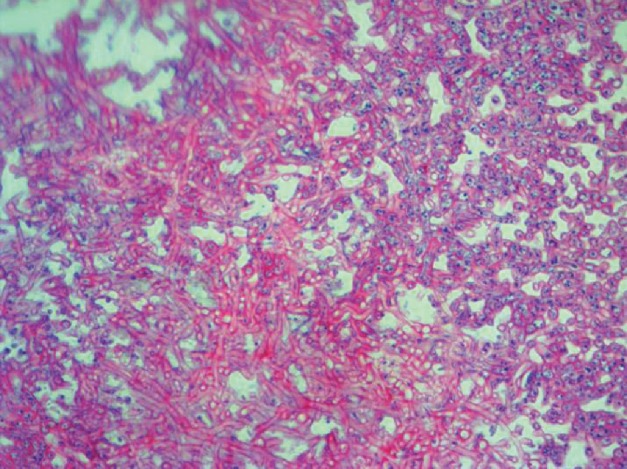
A histopathological study revealed large and broad nonseptal hyphae branching of Mucorales.

**Figure 3:**
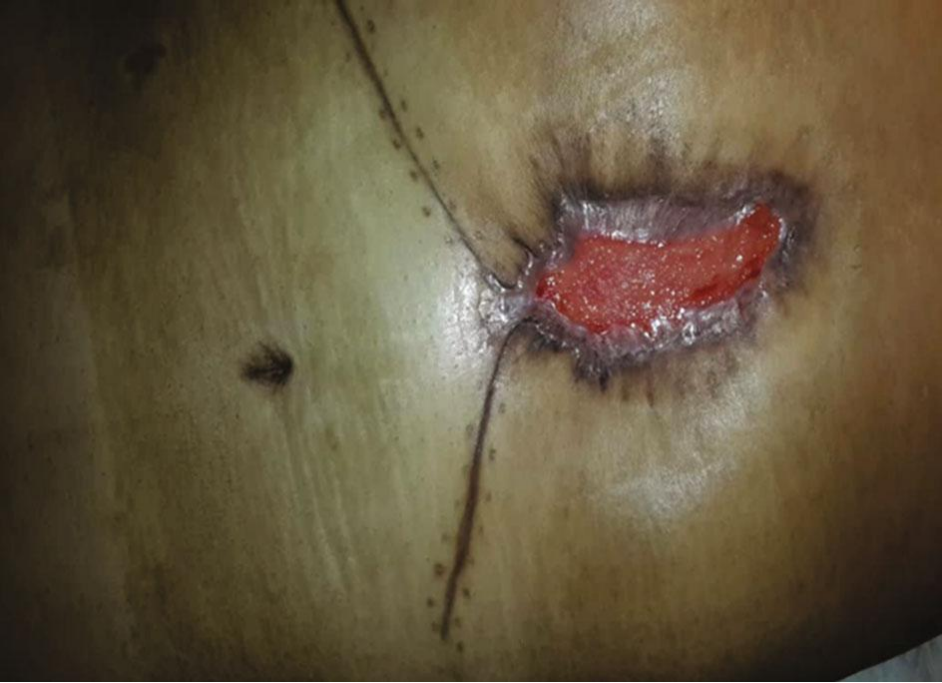
Follow-up at the time of discharge after 2 months.

Standard immune suppression regimen in our center is methylprednisolone, mycophenolate mofetil (CellCept) and tacrolimus (Prograf); however, because of renal failure, he received antithymocyte globulin (ATG) instead of methylprednisone. The patient was transferred to the ward on the 5^th^ day of transplantation. On the 8^th^ day after transplantation, the patient's body temperature raised to 38°C without any other signs or symptoms except purulent discharge from the surgical site. Sepsis work-up was done (all laboratory tests briefly shown in Table 1), and smear and culture from wound discharge were sent for bacterial and fungal pathogens. He received empirical antibiotics (meropenem and vancomycin). Chest X-ray (CXR) was normal, and no collection was reported in the abdominopelvic sonography. The color of wound trended to black in the next day, and necrosis was seen in the surgical site (Figure 1). A microbiological study showed mycelium in wound culture on day 10, and liposomal amphotericin B was started and the dose of immunosuppressive agents was reduced. Furthermore, the smear of the discharge was positive for aseptate hyphae, and the report of fungal culture revealed *Rhizopus* sp. In the histopathological examination, mucormycosis was confirmed too (Figure 2). Spiral chest CT scan and paranasal CT were normal. Repeated surgical debridement was done until reporting a normal histopathological examination on the debrided skin and soft tissues. Following surgery, we used Vacuum-Assisted Closure for the treatment of the surgical site and it resulted in relative remission. At the time of discharge, the wound improved significantly (Figure 3) and we prescribed liposomal amphotericin B for 2-3 times a week until the complete remission. His wound completely improved during the last follow-up visit on the 9^th^ month of LT.

## 3. Discussion

The cutaneous mucormycosis typically is a rare condition; however, it has a good survival rate when diagnosed early [3, 4]. Although definite prevalence of skin mucormycosis is unknown, according to the review of previous studies, it is estimated that this disease has a prevalence of 0.43 to 1.7 per million [7]. Inhalation or inoculation of spores of Mucorales on the skin or mucous is the common transmitted pathway in humans [8]. Past reports suggested that the most frequently isolated strains of cutaneous mucormycosis are Rhizopus oryzae, Lichtheimia corymbifera, and Apophysomyces elegans [9]. Recently, a case report showed a new species called Apophysomyces mexicanus [10]. Our case is infected by *Rhizopus sp.*; however, because of the laboratory limitations, we cannot determine the subspecies of the agent. In the study of 929 cases of mucormycosis, 176 (%18) individuals had skin involvement [11]. The most infected areas are the arms and legs, and other locations include the thorax, back, abdomen, and perineum [12]. Wound infection with mucormycosis like our case has been reported by Ahmadinejad et al. in 2013 that was a case of a diabetic patient with rapidly progressive skin and deep soft tissue necrosis of the hand and forearm after traditional dressing. The clinical presentation varies and depended on patients' immune response condition; typically, initial presentation includes indurated plaques that are erythematous purple, and they become necrotic with an erythematous halo and change into a scar [13]. Other presentations included targeted lesions, tender nodules, ulcers, purpuric lesions, and swollen and scaly plaques [14]. Cutaneous mucormycosis in our patient was presented as a necrotic and black scar in the surgical site early after LT.

In addition to those of immunocompromised patients, there are other risk factors such as diabetes mellitus, dirty crash injury ulcers, scorch wound, undernourishment, use of broad-spectrum antibiotic, consumption of deferoxamine, intravenous drug use, and low birth weight [15].

In liver transplant patients, the known risk factors for fungal infections are steroid use after transplant, prolonged duration of transplant or reoperations, infectious syndrome especially bacterial infections, and use of antibiotics in the posttransplant phase [16]. The length of operation of our patient was not prolonged, and he did not have confirmed bacterial infection. However, our patient had renal failure, a known risk factor of invasive fungal infection after liver transplantation [17].

The early diagnosis and treatment of this disease are important especially in the high-risk group patients and have influence on the good prognosis of affected patients including patients with cutaneous mucormycosis. Thus, examination of histopathology, direct biopsy of wound, and also wound culture should be evaluated to detect fungus. Fungal cultures are positive in 50% of cases, but in recent reviews, there has been a clear increase in culture positivity from 72% to 89% in cutaneous locations due to the development of techniques of cultures [9]. In this report, wound culture and direct histopathological study from wound biopsy were useful for correct diagnosis.

Amphotericin B remains the gold standard treatment of zygomycosis. Because of good tolerability and less side effects of lipid formulations of amphotericin B, it is a better choice. It is also able to deposit into the reticuloendothelial system, including local sites of infection [14]. Vacuum-Assisted Closure (VAC) is a technique which helps in improving surgical site infection resistant to treatment. It has been extensively used in infected and postoperative wounds [15]. Furthermore, Vacuum-Assisted Closure was helpful for healing our patient's surgical site too.

The prognosis of mucormycosis differs in different localizations and host conditions; however, even in the recent years, the mortality rates are between 4 and 10% for localized cutaneous disease [9]. However, Haque et al. reported 33% mortality in their review. Mortality was higher in patients with a delay in diagnosis and treatment. They reported a case of surgical site mucormycosis infections in a liver transplant recipient and reviewed 15 previously reported cases in which 9 of the cases were after liver transplantation [18]. Fortunately, our patient is alive now with complete remission of his wound.

The present case confirms that cutaneous mucormycosis after liver transplant might have a good prognosis with early diagnosis and proper and effective treatment. Combination of antifungal and surgical debridement is the useful treatment for cutaneous mucormycosis. In conclusion, in addition to those of an immunocompromised patient, such as solid organ transplantation, surgical site infection with fungal pathogens should be evaluated, since postponing diagnosis may lead to poor outcome.

## Figures and Tables

**Table 1:** Laboratory tests.

Tests	Day 1	Day 2	Day 3	Day 4	Day 5
WBC	8700	15100	8200	5900	9800
HB	13	13.9	10	9.4	12
PLT	62000	102000	19000	26000	89000
Cr	0.8	1.4	2.5	2.1	1.2
AST	417	274	118	84	53
ALT	183	161	117	91	105
ALP	188	124	153	—	287
BIL(t)	5.8	7.2	1.5	1.1	—
BIL(D)	2.1	4.1	1	0.7	—
INR	4.4	2.9	1.6	1.4	—
ESR	10				
CRP	56				
B/C ^∗^2	NEG				

Abbreviations: WBC: white blood cells (cells/mcl); Hb: hemoglobin (gr/dl); PlT: platelets (n/ml); Cr: creatinine (mg/dl); AST: aspartate aminotransferase (Iu/l); ALT: alanine aminotransferase (Iu/l); ALP: alkaline phosphatase (Iu/l); BIL(t): bilirubin total (mg/dl); BIL(D): bilirubin direct (mg/dl); INR: international normalized ratio; ESR: erythrocyte sedimentation rate; CRP: C-reactive protein; B/C: blood culture.
